# Capsiate attenuates atherosclerosis by activating Nrf2/GPX4 pathway and reshaping the intestinal microbiota in ApoE^−/−^ mice

**DOI:** 10.1128/spectrum.03155-24

**Published:** 2025-03-03

**Authors:** Yongbin Shen, Chuanqi Zhang, Xue Jiang, Xianwei Li, Bo Chen, Weiliang Jiang

**Affiliations:** 1 Department of Vascular Surgery, The 2nd Affiliated Hospital of Harbin Medical University 105821 , Harbin, China; Jilin University, Changchun, China

**Keywords:** atherosclerosis, capsiate, TRPV1, gut microbiota, ferroptosis

## Abstract

Atherosclerosis (AS) is the basis of cardiovascular diseases (CVDs) and remains the major contributor to death worldwide. Capsiate is derived from sweet pepper fruit and exhibits numerous pharmacological activities. The objective of this study was to elucidate the protective role of capsiate in atherosclerosis by examining its effect and the underlying regulatory pathways. Here, we showed that capsiate treatment alleviates atherosclerosis in atherosclerosis-prone apolipoprotein E-deficient (ApoE^−/−^) mice. We found that capsiate effectively reduced the plaque area and body weight compared to the Model group. Capsiate inhibited inflammatory response by downregulating phosphoinositide 3-kinase/protein kinase B/nuclear factor-κB pathway. Additionally, further investigation indicated that capsiate could regulate lipid levels in mice via reducing the expressions of 3-hydroxy-3-methylglutaryl coenzyme A reductase and low-density lipoprotein receptor, and increasing the expression of recombinant cytochrome P450 7A1. Furthermore, capsiate effectively activated transient receptor potential vanilloid subfamily member 1 in ApoE^−/−^ mice fed a high-fat diet. The microbial sequencing demonstrated capsiate administration significantly regulated the gut microbiota disturbance and increased some beneficial bacterial (*Lachnospiraceae NK4A136* group) levels in ApoE^−/−^ mice. Human umbilical vein endothelial cells (HUVECs) were exposed to oxidized low-density lipoprotein (ox-LDL) to stimulate atherosclerotic endothelial damage *in vitro*. Our study revealed that capsiate inhibited ox-LDL-induced HUVECs injury and inflammation. We further investigated the effects of capsiate on ferroptosis *in vivo* and *in vitro*; it was found that capsiate exhibited anti-ferroptosis through regulating nuclear factor erythroid 2-related factor 2/glutathione peroxidase 4 pathway. Interestingly, ML385 reversed the anti-ferroptosis effect of capsiate in HUVECs. Taken together, our findings suggest a promising use of small-molecule drug capsiate for the treatment of AS and related CVDs.

**IMPORTANCE:**

Capsiate has been found to inhibit fat accumulation, promote energy metabolism, and exhibit anti-inflammatory and antioxidative properties. However, there has still been no study on the ferroptosis and gut microbiota of capsiate in atherosclerosis (AS) mouse models. Our study is the first to report on the reshaping of the structure of the gut microbiota by capsiate in AS, and to explore the potential mechanism underlying the improvement of AS. In this study, we demonstrated that capsiate could effectively alleviate high-fat diet-induced AS in apolipoprotein E-deficient mice by inhibiting inflammatory response, improving serum lipid profiles, activating transient receptor potential vanilloid subfamily member 1 pathway, and suppressing ferroptosis. Moreover, the study reported the potential of gut microbiota as mediators of capsiate therapy for AS in animal models. Therefore, these findings may provide robust experimental support for the clinical use of capsiate for AS treatment.

## INTRODUCTION

Atherosclerosis (AS), marked by plaque buildup in arteries, is a chronic inflammatory vascular disease. AS is characterized by inflammatory cell infiltration, lipid deposition, plaque rupture, and thrombus formation as its main features, which is the leading cause of cardiovascular disease (CVD)-related illness and death worldwide. Various pivotal mechanisms have been identified such as endothelial cell damage, lipid metabolic aberrations, and oxidative stress (1, 2). At present, inhibitors of the peptidase proprotein convertase subtilisin/kexin type 9 (PCSK9) and statins are widely recommended in clinical practice to alleviate AS (3). However, the role of these drugs in AS is limited, and they have strong adverse effects, such as increased significant risk of developing cataracts and diabetes, renal dysfunction, as well as muscular side effects (4, 5). Therefore, more effective treatment strategies and means with fewer side effects are urgently needed.

Multiple studies have confirmed that inflammatory response and inflammatory injury play important roles in all stages of atherogenesis (6). Nuclear factor (NF)-κB is a transcription factor in the regulation of multiple adhesion molecules and the response process (7). It has been shown that NF-κB could be regulated via the phosphoinositide 3-kinase (PI3K)/protein kinase B (AKT) (8). The PI3K/AKT pathway is associated with adhesion, invasion, proliferation, metabolism, and angiogenesis (8). In recent years, there have been many studies on PI3K/AKT/NF-κB as targets for AS therapy (9, 10). Researchers have found a strong correlation between intestinal flora changes and the occurrence of AS (11). The intestinal microbiota is crucial in modulating the functions of the immune system. In a previous study, Dingxin Recipe IV decreased the relative abundance of *Erysipelotrichaceae*, increased the abundance of *Muribaculaceae*, and alleviated AS in apolipoprotein E-deficient (ApoE^−/−^) rats by regulating the composition of the gut microbiota (12). Research has shown that the abundance of *Bacteroides* reduces among the intestinal microorganisms of patients with coronary AS, while the abundance of *Firmicutes* increases (13). Furthermore, intestinal microbiota can relieve AS in mice by regulating bile acid metabolism (14). Despite the lack of clarity regarding the underlying mechanisms involved, these results indicate that gut microbiota may contribute to the development of AS. Thus, regulating the homeostasis of the gut microbiota is a new strategy for the prevention of AS.

Recent animal and clinical studies have confirmed that ferroptosis is an important regulatory factor for various metabolic diseases (15, 16). Ferroptosis, a form of regulated cell death, relies on excessive lipid peroxidation and iron (17). The main pathogenic mechanisms of ferroptosis are associated with iron metabolism disorders under the excessive accumulation of reactive oxygen species (ROS). Previous studies showed that high-fat diet can trigger ferroptosis, which plays a crucial role in AS and non-alcoholic fatty liver (18, 19). Glutathione peroxidase 4 (GPX4) is considered a primary antioxidant enzyme and GPX4/SLC7A11 axis is one of the pathways to inhibit ferroptosis (20). Inactivation of GPX4 led to excessive lipid peroxidation and ROS accumulation, thus triggering ferroptotic cell death. In addition, in the context of cellular defense against ferroptosis, nuclear factor erythroid 2-related factor 2 (Nrf2) plays a pivotal role in regulating antioxidant responses and many crucial metabolic pathways, including lipid metabolism, proteolysis, iron metabolism, and apoptosis (21). Previous studies demonstrated that Nrf2 regulates the transcription of SLC7A11, thereby activating expression of the downstream protein GPX4 (22). Hence, accumulating evidence suggests that targeting Nrf2/GPX4 signaling route may inhibit ferroptosis, potentially decelerating the development of AS.

**Fig 1:**
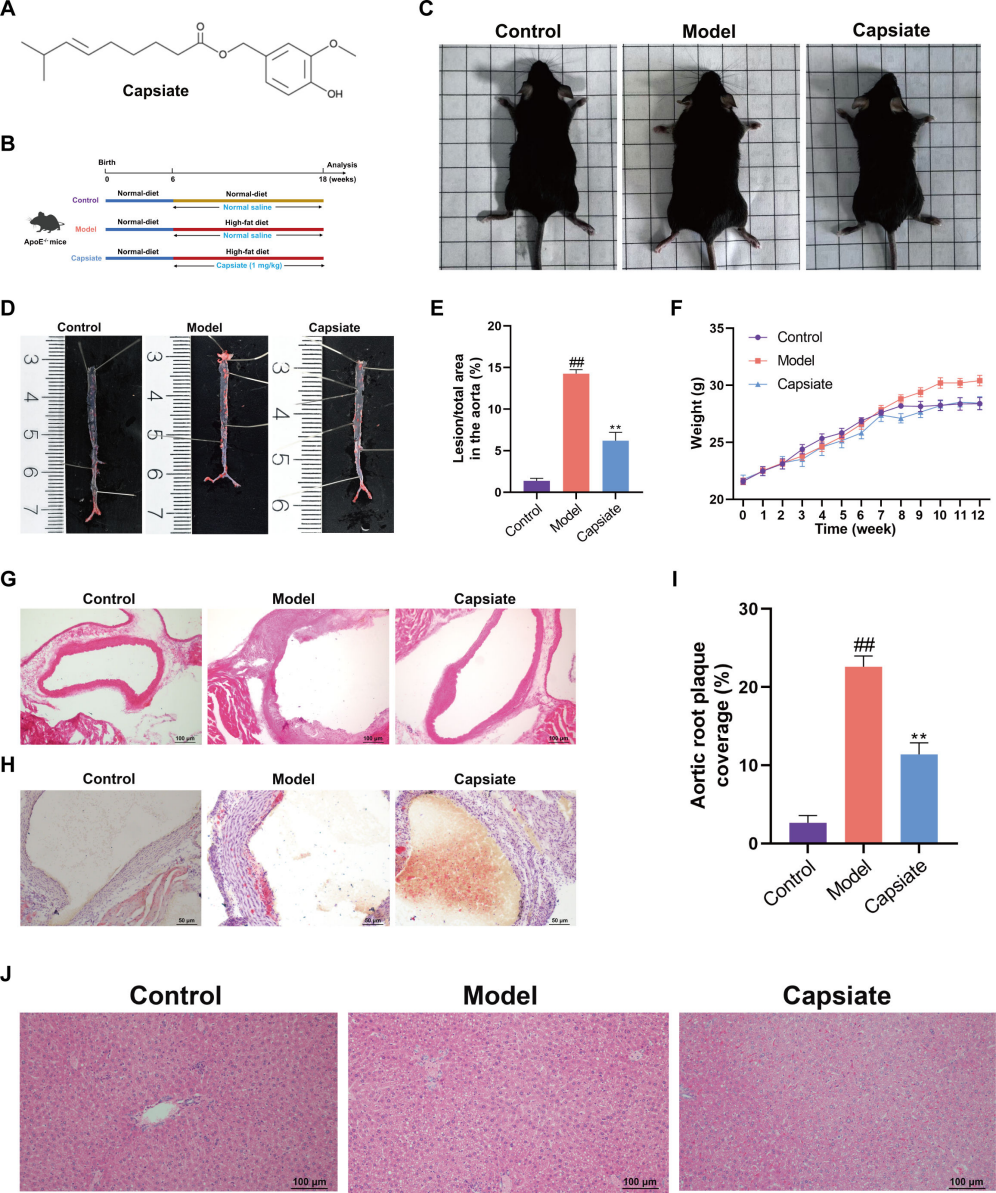
Capsiate alleviates atherosclerosis in high-fat diet-fed ApoE^−/−^ mice. (**A**) The molecular structure of capsiate. (**B**) The study design scheme illustrates groups with treatment. (**C**) General state of mice. (**D**) Oil Red O staining of the whole aorta of ApoE^−/−^ mice (*n* = 3). (**E**) Quantification of the lesion area stained with Oil Red O (*n* = 3). (**F**) Weekly monitoring of mouse body weights and plotting of line graphs (*n* = 10). (**G**) Hematoxylin and eosin (H&E) staining of atherosclerotic plaque lesions in the aortic arch (*n* = 3). Scale bars: 100 µm. (**H**) Oil Red O staining on aortic root sections from the indicated mice (*n* = 3). Scale bars: 50 µm. (**I**) H&E staining quantitative analysis of aortic sinus lesions (*n* = 3). (**J**) Liver H&E staining (*n* = 3). Scale bars: 100 µm. Data are the mean ± SEM. ^##^*P* ˂ 0.01versus the Control group; **P ˂ 0.01 versus the Model group.

Chili peppers are widely consumed by individuals globally. They not only enhance the flavor and spiciness of culinary dishes but also exhibit potential medicinal properties (23). The introduction of capsiate (Fig. 1A), a newly discovered non-pungent analog of capsaicin, offers a potential alternative for individuals who avoid foods containing capsaicin due to its pungent properties (24). Currently, capsaicin has been shown to ameliorate high-fat diet-induced AS by activating transient receptor potential vanilloid subfamily member 1 (TRPV1) and remodeling gut microbiota (25, 26). Capsiate and capsaicin exhibit analogous chemical structures; however, the pronounced pungency and tendency to induce gastrointestinal adverse effects have constrained their application in clinical trials. In contrast to capsaicin, capsiate demonstrates a reduced level of irritancy while exhibiting comparable or potentially superior biological activities, including enhancements in insulin sensitivity and improvements in lipid and glucose metabolism (27, 28). Research has indicated that the reduction of body fat in humans due to capsiate is linked to the activation of thermogenic processes in brown adipose tissue (29). Nevertheless, the effects of capsiate on the progression of AS have yet to be documented in the literature.

This study was the first to examine if capsiate could mitigate AS under a high-fat environment *in vivo* and *in vitro*. We also sought to ascertain the exact mechanism of capsiate in inflammatory response, lipid metabolism, and oxidative stress. The principal outcomes of this research indicated that capsiate effectively diminished lipid accumulation, suppressed inflammatory responses, and inhibited ferroptosis. Furthermore, our investigation demonstrated that the influence of capsiate on the composition of gut microbiota is associated with its pharmacological effects in the management of AS.

## MATERIALS AND METHODS

### Chemicals and reagents

Capsiate (99% pure) and ML385 (99% pure) were purchased from MedChemExpress (Monmouth Junction, NJ, USA). Glutathione (GSH), triglyceride (TG), and lipid peroxide (LPO) assay kits were purchased from the Jiancheng Bioengineering Institute (Nanjing, China). Total cholesterol (TC) and low-density lipoprotein cholesterol (LDL-C) assay kits were obtained from Yeasen Biotechnology (Shanghai, China). Oil Red O (ORO), iron, malondialdehyde (MDA), and superoxide dismutase (SOD) assay kits were purchased from Solarbio Science & Technology Co., Ltd. (Beijing, China). ROS assay kit and enzyme-linked immunosorbent assay (ELISA) kits for mouse Tumor Necrosis Factor-αlpha (TNF-α), interleukin (IL)-6, IL-1β, and IL-10 were obtained from Ebioscience (Wuhan, China). ELISA kits for human IL-6 and IL-1β were purchased from Jianglai Biological Industrial Co., Ltd. (Shanghai, China). Human TNF-α ELISA kit was purchased from ABclonal (Boston, MA). Antibodies against TRPV1, p-NF-κB, NF-κB, β-actin, Nrf2, GPX4, and cytochrome P450 7A1 (CYP7A1) were purchased from Cell Signaling Technology Inc. (Boston, MA, USA). Antibodies against PI3K, p-AKT, AKT, SLC7A11, 3-hydroxy-3-methylglutaryl coenzyme A reductase (HMGCR), and low-density lipoprotein receptor (LDLR) were purchased from Abcam Inc. (Cambridge, UK).

### Animals models

A total of 30 male 5-week-old ApoE^−/−^ mice were generated and acquired from Beijing Weitong Lihua Experimental Animal Technology (Beijing, China). All mice were housed under standard laboratory conditions with a 12 h/12 h light/dark cycle, and a constant temperature of 22 ± 2 ℃. After 1 week of adaptive feeding, 6-week-old ApoE^−/−^ mice were randomly divided into three groups (*n* = 10) and treated as follows (Fig. 1B): ApoE^−/−^ mice were fed the standard diet (Control group); ApoE^−/−^ mice were fed high-fat diet (Model group); ApoE^−/−^ mice were fed high-fat diet plus capsiate at a dose of 1 mg/kg/day (Capsiate group). The dosage and administration method of capsiate were determined based on earlier research studies (30, 31). Mice in the Capsiate group received capsiate through intraperitoneal injection for 12 weeks, while mice in the other groups received equal normal saline by the same route. Throughout the experimental period, mice were fed freely while their body weights were measured weekly. At the end of 18 weeks, all animals were anesthetized using an intraperitoneal injection of sodium pentobarbital at a dosage of 50 mg/kg body weight and then euthanized by cervical dislocation. Plasma samples were subsequently collected and immediately frozen. The hearts and aortas were removed carefully and fixed with 4% paraformaldehyde at 4°C for 24 h or preserved in optimal cutting temperature compound for subsequent staining procedures.

### Evaluation of atherosclerotic lesions

To investigate the formation of atherosclerotic plaques *in vivo*, a laparotomy was performed on the mice along the midline of the abdomen up to the sternal angle, followed by the excision of the heart, liver, and aorta. Three whole aortas were collected from each experimental group, and gross specimens were prepared using Oil Red O staining. In summary, the aortas were preserved in 4% paraformaldehyde, followed by three washes with phosphate buffer saline (PBS) to eliminate any excess paraformaldehyde. The samples were then soaked in 60% isopropanol for 10 minutes and stained with a freshly made Oil Red O working solution for 30 minutes. After differentiating in 60% isopropanol until the lipid plaques in the lumen turned orange-red or bright red and the arterial walls became almost transparent, the vessels were opened longitudinally with dissecting scissors, and the arterial walls were secured with fine needles for photography against a black background. The remaining hearts were promptly detached from the ascending aortic root and fixed in a 4% paraformaldehyde solution for universal tissue fixation. The leftover aortic tissue was stored at −80°C for future analyses. The area of atherosclerotic lesions was quantified using ImageJ software, analyzing three cross-sections per mouse (*n* = 3).

### Histopathological analysis

The heart and liver specimens were embedded in paraffin wax, and sections of the aortic sinus and liver tissues were prepared. Hematoxylin and eosin (H&E) staining (Solarbio) was employed to assess the presence of plaques in the aortic root sections across various groups, adhering to the manufacturer’s guidelines. In brief, the prepared sections were soaked in Harris hematoxylin staining solution for 5 minutes, washed with tap water, treated with a differentiation solution, rinsed again with tap water, counterstained with a bluing reagent, and then washed with running water. Next, the sections were dehydrated using a gradient ethanol series of 85% and 95% for 5 minutes each, followed by staining with eosin solution for 5 minutes. After dehydration, the sections were mounted using neutral resin. H&E staining effectively highlights the extracellular lipid composition, including cholesterol crystals and cholesterol esters, as well as the morphology of the lesions. Additionally, Masson staining was conducted to evaluate the collagen content, following the instructions provided in the kit (Solarbio). For immunohistochemistry (IHC) analysis, the paraffin sections were deparaffinized to water, followed by antigen retrieval using citric acid solution, and blocking of endogenous peroxidase with Endogenous Peroxidase Blocking Buffer (Beyotime). After incubation with HMGCR antibody and secondary antibody, the sections were stained with 3,3′-diaminobenzidine (DAB) stain and hematoxylin. All tissue samples were examined and photographed using an optical microscope (Olympus, Tokyo, Japan).

### Cell culture and treatments

Human umbilical vein endothelial cells (HUVECs) were procured from the Shanghai Institute of Chinese Academy of Medical Sciences and subsequently cultured in Dulbecco’s Modified Eagle’s Medium containing 10% fetal bovine serum. The cultures were maintained in a humidified environment with 5% CO_2_ at a temperature of 37°C. In order to replicate endothelial cell injury, oxidized low-density lipoprotein (ox-LDL) at a concentration of 100 µg/mL (Sigma, Louis, MO, USA) was administered to HUVECs for the subsequent cellular experiments. In addition, to confirm whether capsiate exerted its anti-ferroptosis effect by activating Nrf2 pathway, HUVECs were pretreated with 10 µM ML385 for 1 h prior to the administration of capsiate.

### Cell viability

The effect of capsiate on HUVECs was assessed utilizing the methylthiazolyldiphenyl-tetrazolium bromide (MTT) assay (Beyotime Biotechnology, Shanghai, China). In summary, HUVECs were cultured in 96-well plates at a density of 1 × 10^4^ cells per well for a duration of 24 h, followed by treatment with varying concentrations of capsiate (25, 50, 100, 200, and 400 µM) or ox-LDL (25, 50, 100, and 200 µg/mL) for an additional 24 h. For subsequent experiments, HUVECs were exposed to 100 µg/mL of ox-LDL for 24 h and then co-incubated with different concentrations of capsiate (25, 50, and 100 µM) for another 24 h. Following these treatments, the cells were incubated in 100 µL of medium containing 10 µL of MTT reagent at 37°C for 4 h. The absorbance was measured at 570 nm using a microplate reader (BioTek Instruments Inc., Winooski, VT, USA). Each experimental condition was performed in six replicate wells to ensure data reliability.

### Measurement of biochemical indicators

LPO levels in HUVECs were quantified using an LPO assay kit (Jiancheng, Nanjing, China). The ROS levels in arterial tissue were assessed utilizing a commercially available ROS fluorescence assay (Elabscience). Iron concentrations in arterial tissue, as well as the levels of SOD and MDA in both HUVECs and arterial tissue, were measured by commercial kits (Solarbio). The GSH levels in HUVECs were analyzed according to the manufacturer’s instructions using a glutathione assay kit (Jiancheng). The contents of TG and TC were detected by the glycerol-3-phosphate oxidase-phenol-aminophenol (GPO-PAP) method, while the content of LDL-C was measured by the double-reagent method using commercially available kits. All measurements for the respective indices were conducted using a microplate reader.

### Enzyme-linked immunosorbent assay

Following the collection of serum and the supernatant from the medium, the levels of TNF-α, IL-6, IL-1β, and IL-10 were detected by the ELISA kits on the basis of the manufacturer’s instructions. The absorbance at 450 nm was measured by a microplate reader.

### Western blotting

Protein samples were extracted from both cultured cells and tissue samples using radioimmunoprecipitation assay buffer containing protease inhibitors. The quantification of protein levels was carried out using the bicinchoninic acid protein assay kit (obtained from Solarbio, China). Equal amounts of the protein extracts were subjected to electrophoresis on SDS-PAGE gels, followed by transfer to polyvinylidene fluoride sheets. These membranes were subsequently blocked using a 5% solution of bovine serum albumin for 1 h, and then incubated overnight at 4°C with the appropriate primary antibodies. Following this, a 1 h incubation with the corresponding secondary antibodies was performed. The detection of the proteins was conducted using enhanced chemiluminescence Western blotting detection solutions (Solarbio) and a chemiluminescence detection system. The relative expression of proteins was calculated according to the reference bands of β-actin. Band intensities were quantified using ImageJ software, and the results were plotted utilizing the GraphPad Prism 8.0.2 software. This procedure was performed in triplicate.

### RNA isolation and quantitative reverse transcription polymerase chain reaction (qRT-PCR)

Total RNA was extracted from HUVECs with a commercial RNA isolation kit (QIAGEN RNeasy 74106). qRT-PCR was conducted using the HiScript II One-Step qRT-PCR SYBR Green Kit (Vazyme, Nanjing, China), following the manufacturer’s guidelines, on a StepOnePlus Real-Time PCR system (Applied Biosystems, Waltham, MA). A comprehensive list of all primers employed in the study can be found in Table S1. The relative expression levels of gene transcripts were calculated via the 2^-ΔΔCt^ method, with glyceraldehyde-3-phosphate dehydrogenase (GAPDH) serving as the endogenous control.

### DNA extraction and 16S rRNA amplicon sequencing

Fecal samples were taken, promptly frozen in liquid nitrogen, and then kept at −80°C for storage. Using the Fast DNA Stool Mini Kit (QIAGEN, Hilden, Germany), we isolated bacterial genomic DNA from stool samples. The bacterial V3-V4 regions of 16S rRNA genes were amplified using the primer pairs (338F/806R). PCR products were purified using AxyPrep DNA Gel Extraction Kit (Axygen Biosciences, USA). Amplicon pools were prepared for library construction using the Pacific Biosciences SMRTbell Template Prep kit 1.0 (PacBio, USA) and sequenced on PacBio RS II (LC-Bio Technology Co., Ltd., Hangzhou, China). Microbial community analyses, including α-diversity and β-diversity, were conducted utilizing the phyloseq R package. The assessment of α-diversity was performed through the relative inverse Simpson index, while β-diversity was quantified using UniFrac distance. Principal coordinates analysis was employed for ordination purposes. For the differential testing of α-diversity, we utilized the two-tailed Wilcoxon signed-rank test, and *P* < 0.05 was considered statistically significant. Community dissimilarities were evaluated through permutational multivariate analyses of variance with 10,000 iterations. Differentially enriched microbial taxa were identified using Analysis of Composition of Microbiomes with Bias Correction 2 (ANCOM-BC2) (v.2.2.2; default parameters), a methodology for performing differential abundance (DA) analysis of microbiome count data. Differences with fold change >2 and adjusted *P* < 0.05 were deemed statistically significant. Additionally, constrained correspondence analysis was applied to assess microbial dissimilarities in fecal samples.

### Statistical analysis

The data were analyzed using GraphPad Prism version 8.0.2 (GraphPad Software Inc. San Diego, CA, USA). Normally distributed data were presented as the mean ± standard error of the mean (SEM), and group differences were assessed using one-way analysis of variance (ANOVA) and Tukey’s test. Results marked with different letters are significantly different (*P* < 0.05).

## RESULTS

### Capsiate alleviates atherosclerotic progression

At the beginning of the experiment, all subjects exhibited comparable body weights. After a 12 week period during which the mice were administered a high-fat diet, those in the Model group demonstrated a greater degree of obesity compared to the Control and Capsiate groups (Fig. 1C). Additionally, weekly assessments of the body weight changes of ApoE^−/−^ mice were conducted (Fig. 1F). Compared to the Control group, body weight was significantly increased in the Model group. Conversely, the Capsiate group displayed a notable reduction in body weight when compared to the Model group. To evaluate aortic lesion formation after the 12 week intervention,ORO staining was performed on the aortae of the mice (Fig. 1D and E). The Model group presented with a greater number of red plaques in the aorta compared to the Control group, indicating that the excessive fat accumulation induced by the high-fat diet progressively led to the formation of arterial plaques, ultimately resulting in AS. In contrast, the Capsiate group exhibited a significant reduction in both the number and volume of red plaques compared to the Model group, thereby effectively mitigating the risk of vascular obstruction and AS associated with arterial plaque accumulation in the mice. Consistent with these findings, H&E and ORO staining of the aortic sinus revealed a marked alleviation of atherosclerotic lesions in ApoE^−/−^ mice following capsiate intervention (Fig. 1G through I). Long-term high-fat diet not only induces AS in ApoE^−/−^ mice but also contributed to hepatic fat accumulation and subsequent liver injury. H&E staining results indicated the presence of pronounced macrovesicular steatosis in the livers of mice subjected to a high-fat diet compared to their normal counterparts, whereas capsiate treatment significantly ameliorated hepatic steatosis and provided protection to hepatocytes in comparison to the Model group (Fig. 1J). These findings suggest that capsiate effectively inhibits the progression of atherosclerotic lesions and attenuates the development of AS.

### Capsiate attenuates inflammatory responses via the PI3K/AKT/NF-κB pathway in ApoE^−/−^ mice

**Fig 2:**
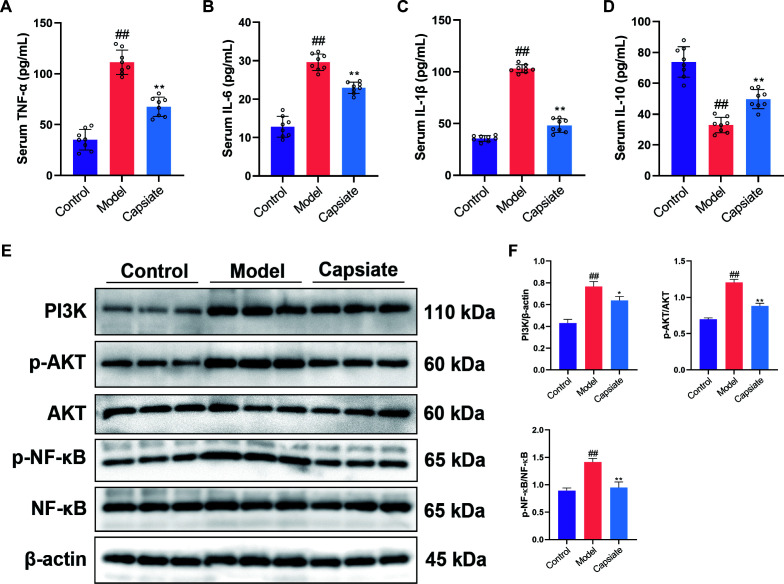
Capsiate inhibits PI3K/AKT/NF-κB pathway in ApoE^−/−^ mice. Enzyme-linked immunosorbent assay assessment of (**A**) TNF-α, (**B**) IL-6, (**C**) IL-1β, and (**D**) IL-10 levels in the serum of ApoE^−/−^ mice (*n* = 8). (**E and F**) The expressions of PI3K, p-AKT, and p-NF-κB were measured by Western blot (*n* = 3). Data are the mean ± SEM. The results are representative of three independent experiments. ^##^*P* ˂ 0.01 versus the Control group; *P ˂ 0.05, **P ˂ 0.01 versus the Model group. The results are representative of three independent experiments.

In order to elucidate the role of capsiate in regulating the inflammatory responses, the plasma levels of four typical inflammatory cytokines, TNF-α, IL-6, IL-1β, and IL-10, were measured by ELISA. Compared with the Control group, the levels of TNF-α, IL-6, and IL-1β were significantly increased in the Model group. Conversely, the administration of capsiate resulted in a reduction of these pro-inflammatory cytokines compared with the Model group. (Fig. 2A through C). Furthermore, the level of IL-10 (anti-inflammatory cytokine) was increased in the serum after capsiate treatment compared to the Model group (Fig. 2D). The NF-κB pathway is one of the main intracellular axes implicated in the activation of the inflammatory response, which can be activated by the PI3K/AKT signaling pathway (8). To illustrate the possible mechanism by which capsiate exerts its effects on inflammation, the PI3K/AKT and NF-κB signaling pathways were detected using Western blotting. As shown in Fig. 2E and F, capsiate treatment suppressed the PI3K/AKT/NF-κB pathway in aortic tissue, as evidenced by the reduced protein expression levels of PI3K, p-AKT, and p-NF-κB when compared to the Model group. These findings suggested that capsiate may mitigate the inflammatory responses in AS model mice through the inhibition of the PI3K/AKT/NF-κB pathway.

### Capsiate attenuates serum lipid accumulation *in vivo*

**Fig 3:**
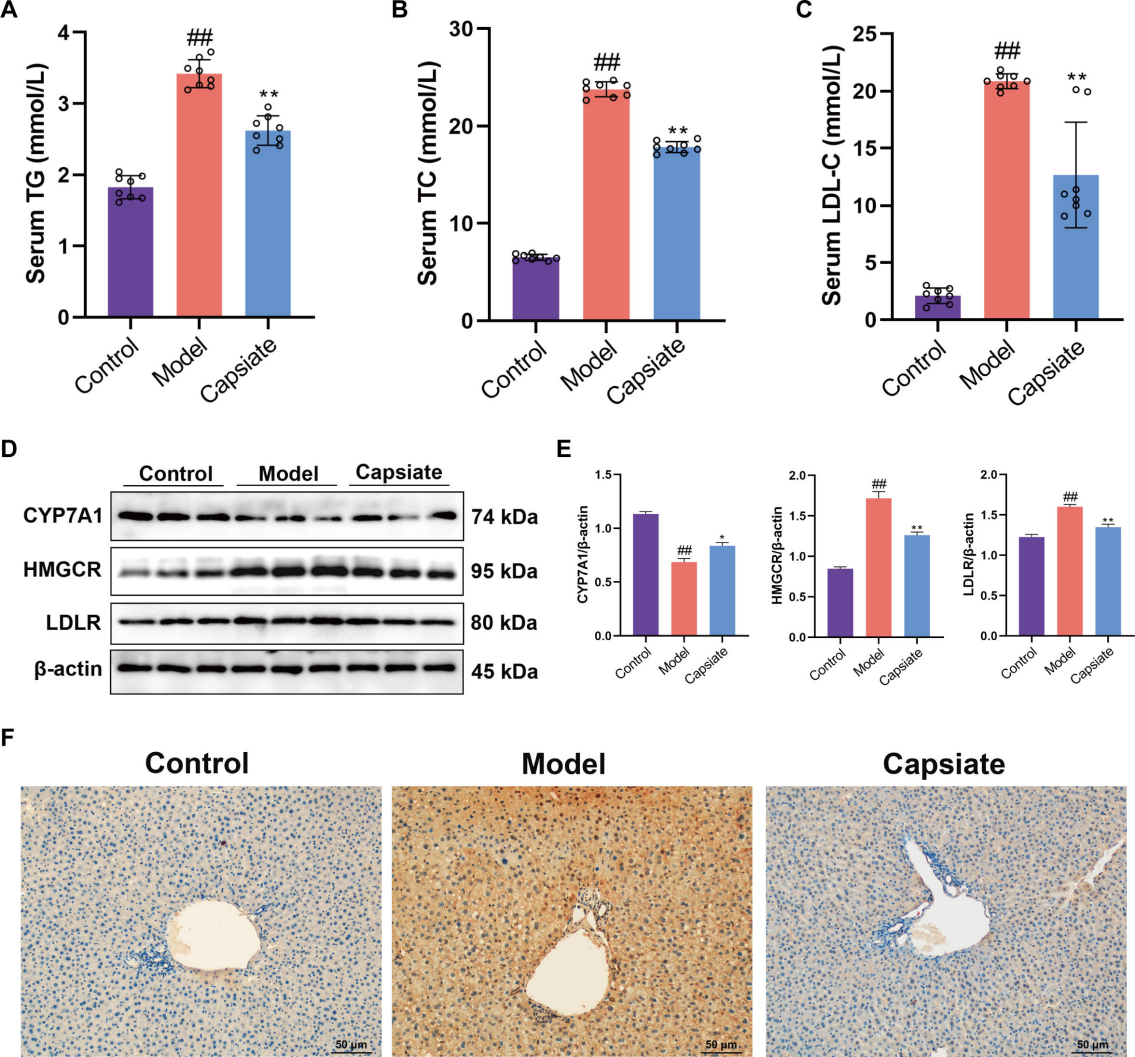
Effect of capsiate on the development of serum lipids in ApoE^−/−^ mice. Concentrations of (**A**) serum TG, (**B**) serum TC, (**C**) serum LDL-C (*n* = 8). (**D and E**) The expressions of CYP7A1, HMGCR, and LDLR were measured by Western blot (*n* = 3). (**F**) Immunohistochemistry of HMGCR was acquired in the indicated groups. Scale bars: 50 µm. Data are the mean ± SEM. The results are representative of three independent experiments. ^##^*P* ˂ 0.01 versus the Control group; *P ˂ 0.05, **P ˂ 0.01 versus the Model group.

It is generally accepted that high levels of lipids in the serum contributed to the development of atherosclerosis (32). In order to evaluate the effects of capsiate on serum lipid in ApoE^−/−^ mice fed high-fat diet, plasma TG, TC, and LDL-C concentrations were measured. Compared with the Control group, levels of TG, TC, and LDL-C were remarkably elevated in the Model group (Fig. 3A through C). However, capsiate significantly inhibited TG, TC, and LDL-C serum levels. The enzyme CYP7A1 is involved in the regulation of bile acid biosynthesis. Western blotting results revealed that capsiate administration led to a marked increase in the protein expression of CYP7A1 when compared to the Model group (Fig. 3D and E). Additionally, HMGCR, a key rate-limiting enzyme for cholesterol synthesis, is subject to regulation through a negative feedback mechanism involving sterols and non-sterol metabolites derived from mevalonate. LDLR plays a crucial role in the endocytosis of LDL-C particles and the maintenance of cholesterol homeostasis. As shown in Fig. 3D and E, a high-fat diet resulted in a significant upregulation of HMGCR and LDLR protein expressions, while capsiate notably inhibited the expression of HMGCR and LDLR. IHC was performed to further assess the levels of HMGCR in the liver. As shown in Fig. 3F, the rates of positive expressions for HMGCR in the Model group were significantly greater than those in the Control group. In contrast, the Capsiate group showed a clear reduction in HMGCR levels compared to the Model group. This was consistent with the observed serum levels of TG, TC, and LDL-C.

### Capsiate prevents AS through activating TRPV1 in high-fat diet-fed ApoE^−/−^ mice

**Fig 4:**
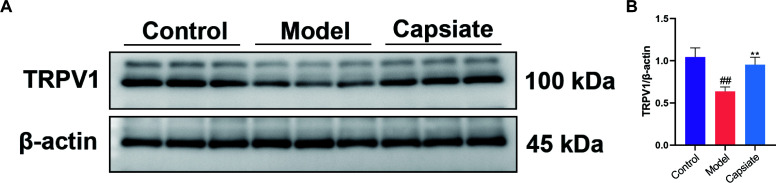
Capsiate attenuates atherosclerosis in ApoE^−/−^ mice via TRPV1. (**A and B**) The expression of TRPV1 was measured by Western blot (*n* = 3). Data are the mean ± SEM. The results are representative of three independent experiments. ^##^*P* ˂ 0.01 versus the Control group; **P ˂ 0.01 versus the Model group.

TRPV1 is a cation channel that is part of the transient receptor potential family. Research indicates that the activation of the TRPV1 channel may mitigate the incidence and progression of CVDs, enhance patient outcomes, and contribute to the protection of the cardiovascular system (33). To investigate whether TRPV1 mediates the effect of capsiate, we assessed TRPV1 expression in ApoE^−/−^ mice. Immunoblotting results showed that capsiate treatment elevated the protein levels of TRPV1 in mice subjected to a high-fat diet combined with capsiate mice, in comparison to those on a high-fat diet alone (Fig. 4A and B).

### Capsiate suppresses ferroptosis in high-fat diet-fed ApoE^−/−^ mice

**Fig 5:**
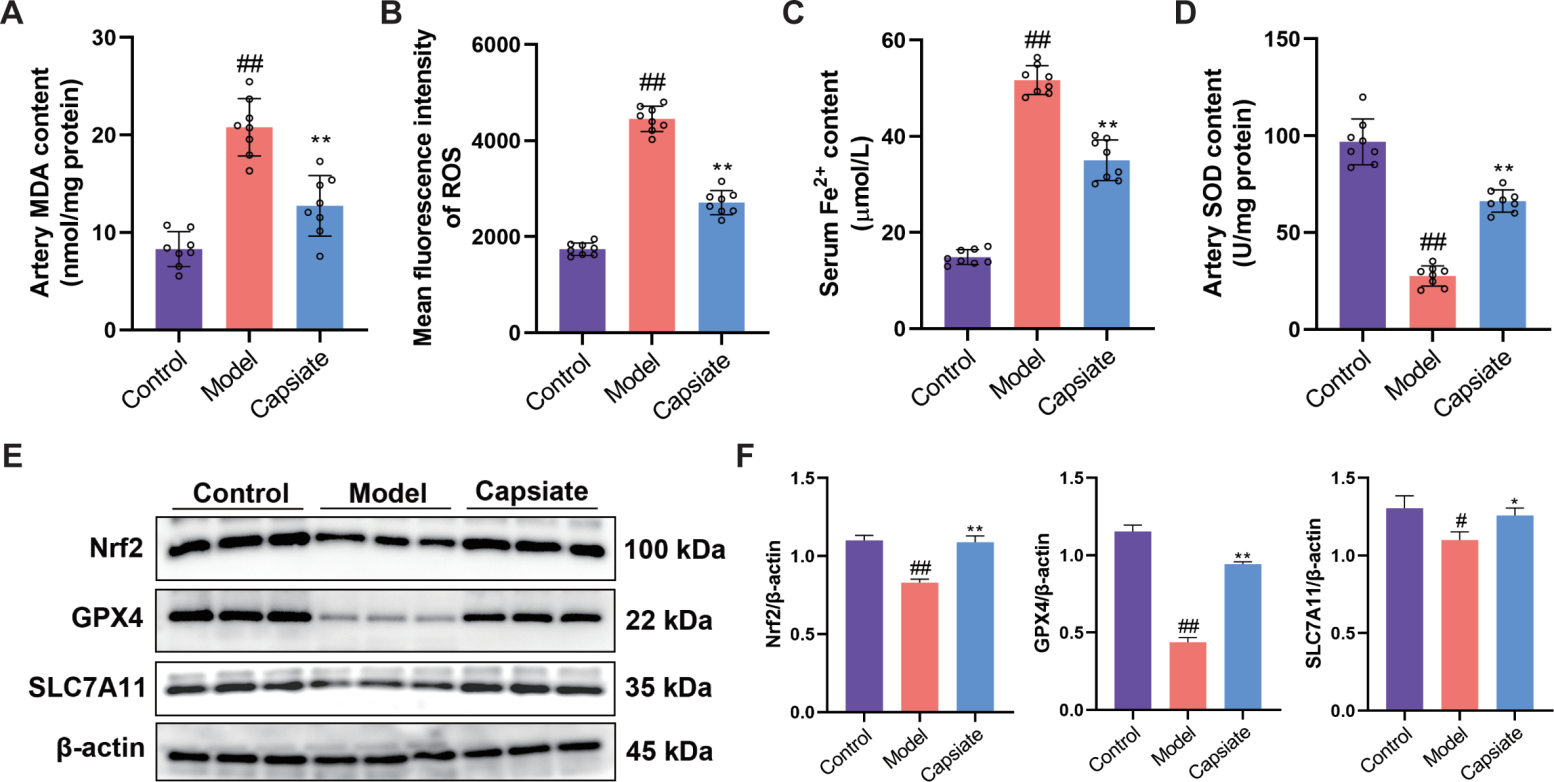
Capsiate inhibits ferroptosis in atherosclerosis. (**A**) MDA, (**B**) ROS, (**C**) iron, and (**D**) SOD levels were detected using the respective kits (*n* = 8). (**E and F**) The expressions of Nrf2, GPX4, and SLC7A11 were measured by Western blot (*n* = 3). Data are the mean ± SEM. A representative example of three independent experiments is shown. ^##^*P* ˂ 0.01 versus the Control group; *P ˂ 0.05, **P ˂ 0.01 versus the Model group.

To explore the potential of Capsiate in preventing ferroptosis in the aortic tissue, we evaluated various indicators associated with ferroptosis. MDA, a byproduct of lipid peroxidation, serves as a biomarker for ferroptosis. Our findings revealed a significant elevation in MDA levels within the arterial lysates of the Model group (Fig. 5A). Additionally, there was a marked increase in both iron and ROS levels, alongside a notable decrease in SOD activity in the aorta of the Model group (Fig. 5B through D). In contrast, following the administration of capsiate, we observed a reduction in MDA, ROS, and iron levels, as well as an enhancement in SOD activity compared to the Model group. Previous studies have reported that Nrf2 regulates transcription of GPX4 and SLC7A11, thereby inhibiting the ferroptosis process (34). Consequently, we hypothesize that capsiate may activate Nrf2 pathway, leading to the upregulation of GPX4 and SLC7A11 expressions in the aorta. Western blot results indicated that high-fat diet resulted in a reduction of the protein levels associated with the Nrf2/GPX4/SLC7A11 signaling pathway in the aorta (Fig. 5E and F). Notably, in the Capsiate group, the expression levels of Nrf2, GPX4, and SLC7A11 were significantly elevated compared to the Model group.

### Capsiate alleviates ox-LDL-induced HUVEC injury

**Fig 6:**
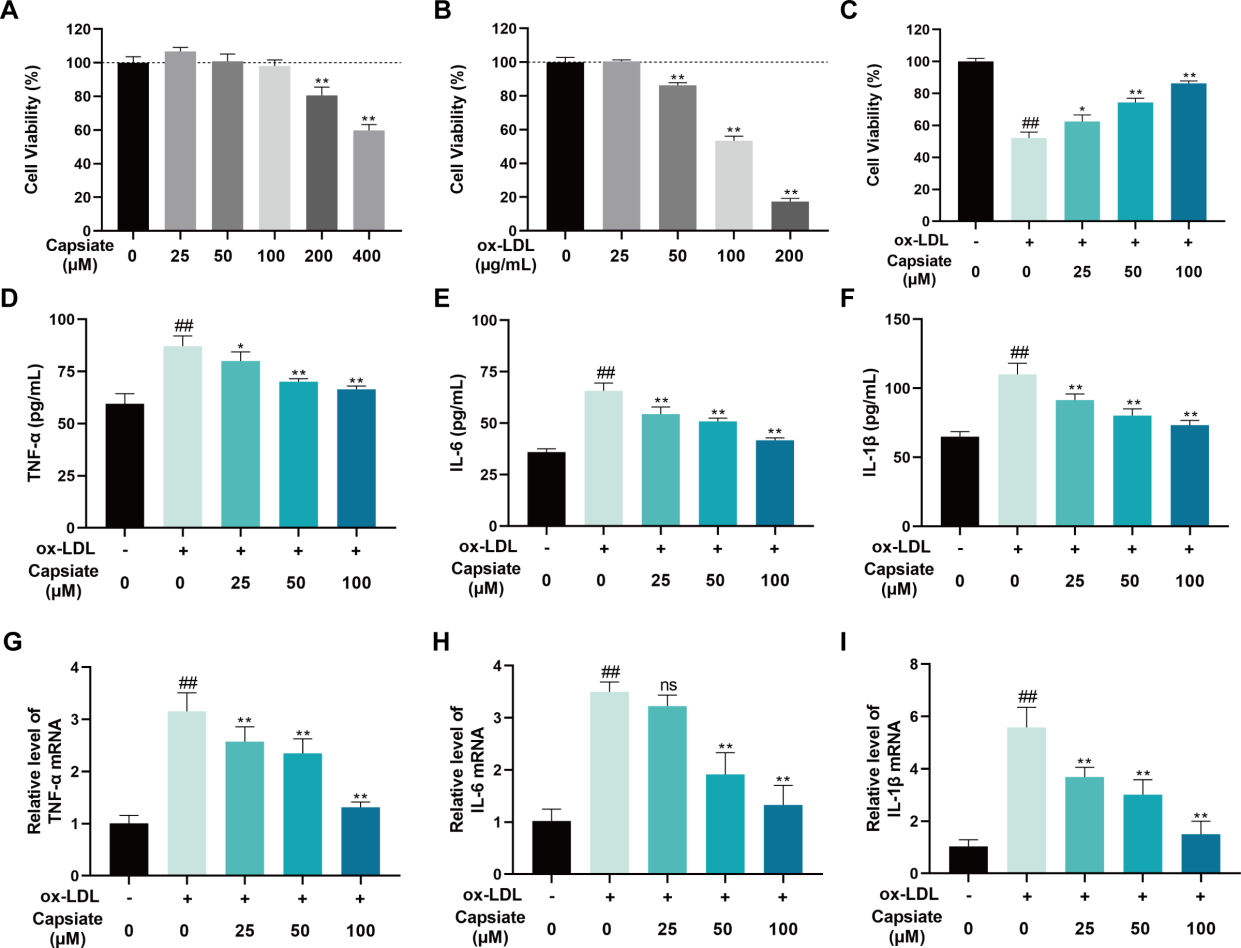
Capsiate inhibits ox-LDL-induced HUVEC injury. Assessing capsiate (**A**) and ox-LDL (**B**) toxicity on HUVECs utilizing MTT assay. (**C**) Assessing the viability and protective impact of capsiate on HUVECs via the use of the MTT assay. (**D–F**) Detection of TNF-α, IL-6, and IL-1β levels was determined within the HUVEC supernatants using commercially respective kits. (**G–I**) mRNA expressions of TNF-α, IL-6, and IL-1β were measured using real-time RT-qPCR. Data are the mean ± SEM (*n* = 6). A representative example of three independent experiments is shown. ^##^*P* ˂ 0.01 versus the Control group; *P ˂ 0.05, **P ˂ 0.01 versus the ox-LDL group. ns, no significance.

To further elucidate the protective effects of capsiate in AS, HUVECs were subjected to ox-LDL to establish an *in vitro* model of endothelial injury. Initially, to measure the cell viability of capsiate on HUVECs, MTT assay was used. The results indicated that capsiate, at concentrations of 25, 50, and 100 µM over a 24 h period, did not exhibit any cytotoxic effects on HUVECs viability (Fig. 6A). In addition, cell death increased with higher concentrations after treatment with ox-LDL (Fig. 6B). The subsequent experiment exposed HUVECS to 100 µg/mL ox-LDL, as it ultimately resulted in approximately a 50% reduction in cell viability. Consequently, these concentrations were selected for further experimentation. The MTT assay results demonstrated a significant reduction in HUVECs viability upon exposure to ox-LDL; however, capsiate treatment significantly improved the viability of ox-LDL-treated HUVECs in a dose-dependent manner (Fig. 6C). In addition, ELISA assay showed that the levels of pro-inflammatory factors (TNF-α, IL-6, and IL-1β) were markedly increased by ox-LDL, which were subsequently reduced following capsiate treatment (Fig. 6D through F). We further analyzed the mRNA levels of pro-inflammatory factors in HUVECs and found that the result is consistent with the changes of protein levels (Fig. 6G through I).

### Capsiate inhibits ox-LDL-induced ferroptosis by activating Nrf2/GPX4/SLC7A11 signaling *in vitro*

**Fig 7:**
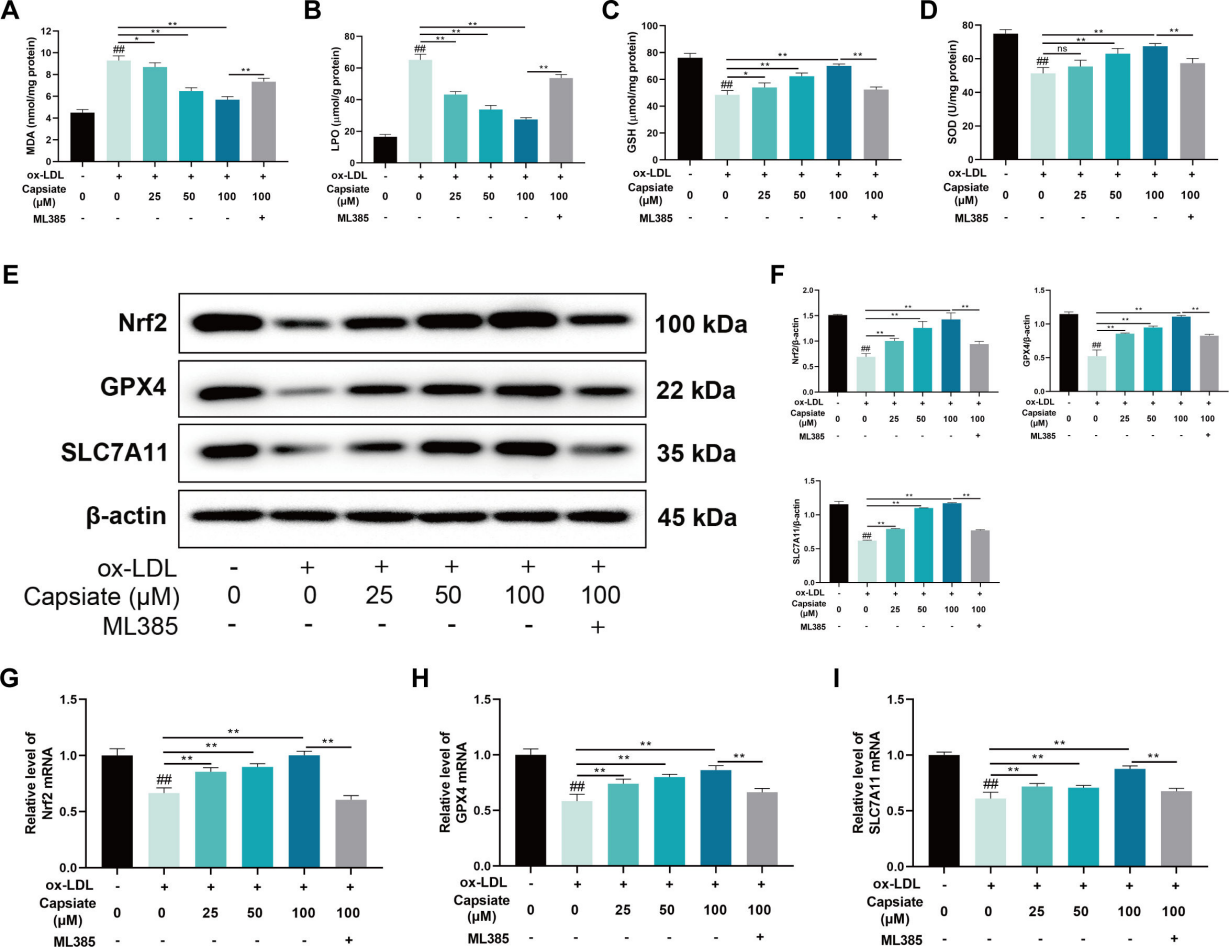
Capsiate suppresses ferroptosis in ox-LDL-induced HUVECs. (**A–D**) The content of MDA, LPO, GSH, and SOD in ox-LDL-treated cells was determined by commercial assay kit (*n* = 6). (**E and F**) The expressions of Nrf2, GPX4, and SLC7A11 were measured by Western blot (*n* = 3). (**G–I**) mRNA expressions of Nrf2, GPX4, and SLC7A11 were measured using real-time RT-qPCR. Data are the mean ± SEM. The results are representative of three independent experiments. ^##^*P* ˂ 0.01 versus the Control group; *P ˂ 0.05, **P ˂ 0.01. ns, no significance.

To further prove the inhibitory effect of capsiate on ferroptosis, the levels of MDA, LPO, GSH, and SOD were determined in HUVECs treated with ox-LDL. The administration of ox-LDL resulted in a significant elevation of MDA and LPO levels in HUVECs, whereas capsiate treatment reduced MDA and LPO levels (Fig. 7A and B). Furthermore, GSH and SOD levels were notably diminished in HUVECs exposed to ox-LDL, whereas capsiate treatment caused an augmentation in antioxidant levels (Fig. 7C and D). Consistent with the *in vivo* results in aorta, capsiate was observed to enhance the protein expressions of Nrf2, GPX4 and SLC7A11 in ox-LDL-induced HUVECs (Fig. 7E and F). The qRT-PCR results revealed that capsiate increased the Nrf2/GPX4/SLC7A11 mRNA levels compared with ox-LDL group (Fig. 7G through I). To ascertain whether the Nrf2 pathway mediates the anti-ferroptotic effects of capsiate, the Nrf2 inhibitor ML385 was employed in HUVECs. The results indicated that HUVECs treated with ML385 effectively counteracted the impact of capsiate on the levels of MDA, LPO, GSH, and SOD in ox-LDL-treated HUVECs (Fig. 7A through D). Importantly, treatment with ML385 significantly inhibited the effect of capsiate on the protein and mRNA expressions associated with the Nrf2/GPX4/SLC7A11 pathway (Fig. 7E through I). These findings suggest that the activation of the Nrf2/GPX4/SLC7A11 pathway may play a critical role in the mechanism by which capsiate inhibits endothelial ferroptosis.

### Capsiate administration altered the diversity of gut microbiota in ApoE^−/−^ mice

**Fig 8:**
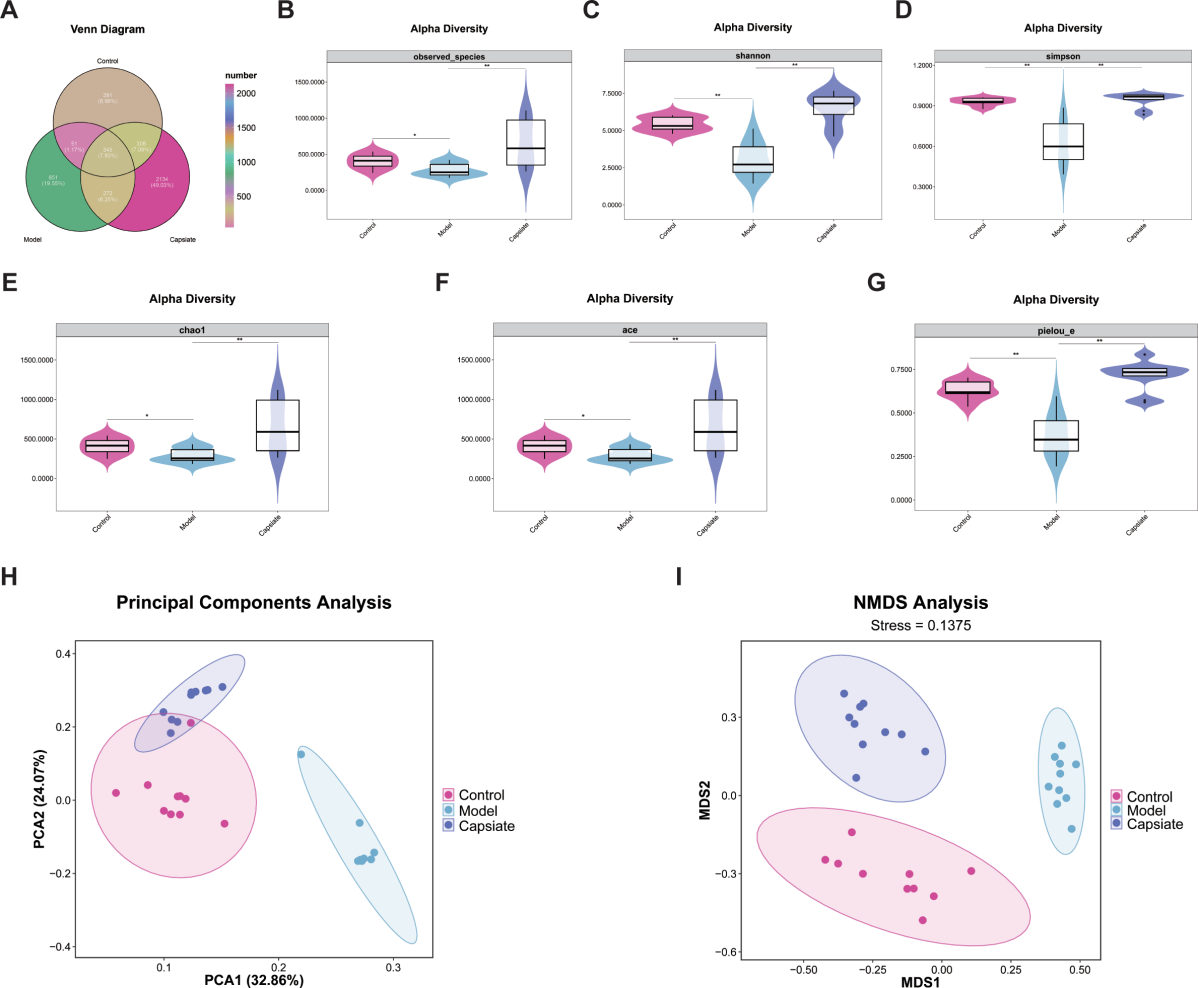
Effects of capsiate on the diversity of gut microbiota in ApoE^−/−^ mice. (**A**) Venn diagrams of bacterial OTUs. (**B–G**) α-Diversity of the gut microbiota assessed by Observed_species, Shannon, Simpson, Chao1, ACE and Pielou_e index. PCA (**H**) and NMDS (**I**) score plots showed the significant differences in the gut microbiota composition. Each data point represents one sample. Data were expressed as the mean ± SEM (*n* = 10). *P < 0.05 and **P < 0.01 compared with the other group.

The gut microbiota is associated with CVDs, including the progression of AS. In this study, we initially examined the diversity of gut microbiota in high-fat diet-fed ApoE^−/−^ mice with or without capsiate treatment by 16S rRNA sequencing. The Venn diagram analysis identified 1,095 operational taxonomic units (OTUs) in the Control group, 1,519 OTUs in the Model group, and 3,059 OTUs in the Capsiate group (Fig. 8A). Notably, the Capsiate group exhibited a greater number of shared OTUs with the Control group compared to the Model group (653 vs 396). The α-diversity indices for the enteric microbiome of the ApoE^−/−^ mice were displayed in Fig. 8B through G. Observed_species, Shannon, Simpson, Chao1, ACE, and Pielou_e index of the Model group were decreased, while capsiate intervention increased the microbiome diversity in feces. To further analyze the structure of gut microbiota, β-diversity based on the principal component analysis (PCA) and non-metric multidimensional scaling (NMDS) were performed. The PCA results indicated a distinct clustering of the Model group compared to the other groups (Fig. 8H) while also revealing overlapping regions between the Capsiate and Control groups. The NMDS analysis indicated that the sample points of each group were largely separated, with the Model group differing significantly from the other groups in terms of flora structure (Fig. 8I). The Capsiate and Control groups demonstrated a high degree of intergroup similarity and relatively similar community composition (Fig. 8H and I). These findings suggest that a high-fat diet reduces the diversity of bacterial communities, whereas capsiate treatment may help maintain the homeostasis of the bacterial community.

### Gut microbiome composition is modulated by capsiate

**Fig 9:**
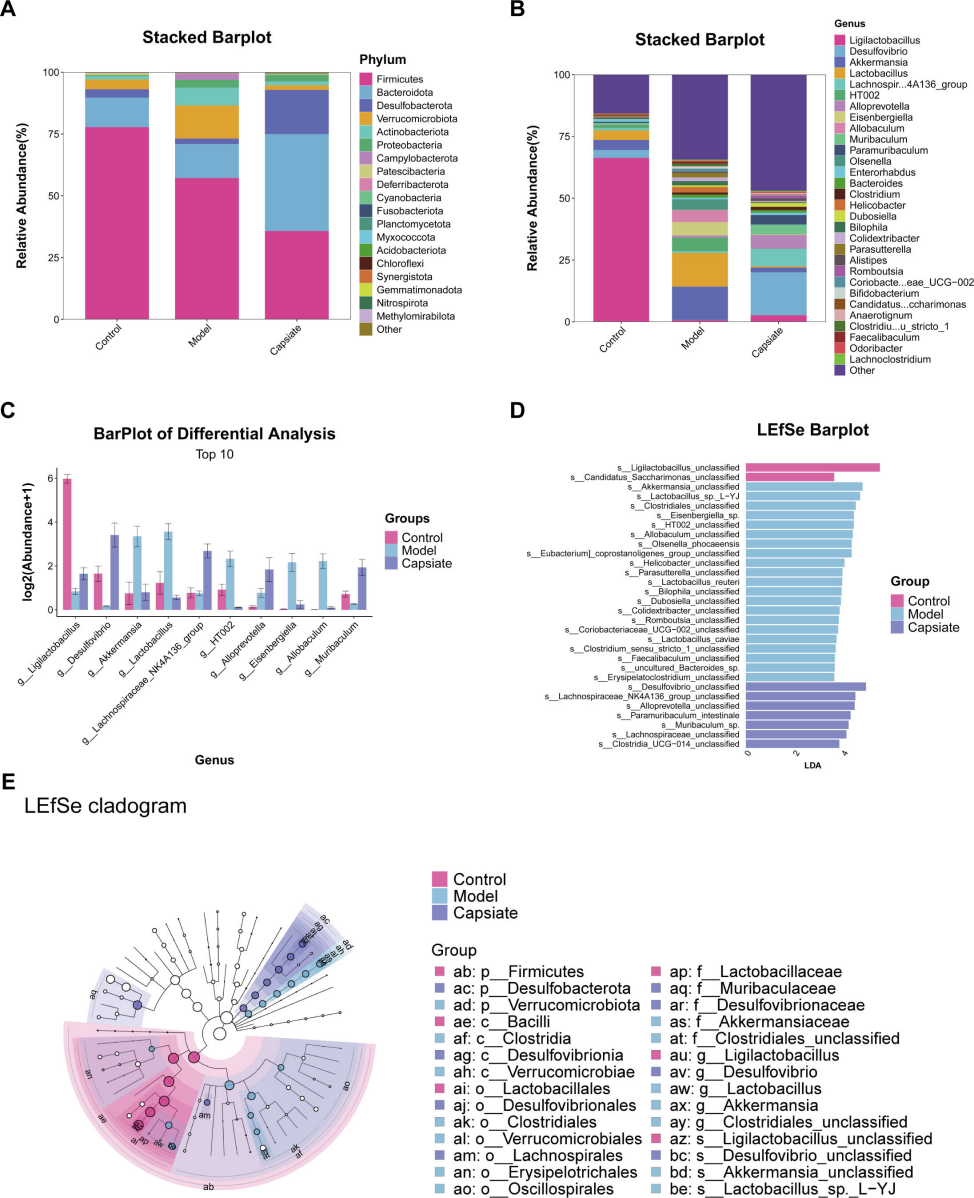
Capsiate significantly altered the composition of gut microbiota in ApoE^−/−^ mice. (**A**) Phylum and (**B**) genus-level community composition. (**C**) Comparison of relative abundance at phylum levels in the three groups. (**D and E**) Cladograms depicting significantly enriched taxa. Differences in the microbiota composition among three groups with LEfSe and LDA >3.

Based on the taxonomic annotation of sequence results, the top 20 most abundant phyla and the top 30 most abundant genera were selected to assess community composition according to intragroup means of relative species abundance (Fig. 9A and B). At the phylum level, a significant increase in the relative abundances of *Verrucomicrobiota* and *Campylobacterota* was observed, while *Desulfobacterota* exhibited a decrease following the administration of a high-fat diet in comparison to the Control group (*P* ˂ 0.05, Fig. 9A). Furthermore, capsiate significantly reduced the relative abundance of *Firmicutes* and *Firmicutes*/*Bacteroidota* (F/B) ratio, but significantly increased the relative abundance of *Desulfobacterota*. An elevated F/B ratio has been associated with gut microbiota dysbiosis and is considered a potential indicator of metabolic disorders (35). Our results show the statistical significance of the gut microbial species of mice in each group at the genus level (*P* ˂ 0.05, Fig. 9B). At the genus level, compared with the Control group, *Akkermansia*, *Lactobacillus,* and *HT002* were the predominant species in the Model group. In addition, the Model group exhibited lower relative abundances of *Ligilactobacillus* and *Desulfovibrio*. Following capsiate intervention, there was a decrease in the relative abundances of *Akkermansia*, *Lactobacillus,* and *HT002*, while the relative abundances of *Desulfovibrio*, *Lachnospiraceae NK4A136*, *Alloprevotella,* and *Muribaculum* increased relative to the Model group. Subsequently, we performed an ANOVA and counted the top 10 different abundant microbiomes at the genus level (Fig. 9C). We found that most of the microbiomes were similar in the Capsiate group compared to the Control group, but there were also some differences in both groups. Capsiate increased the abundance of *Lachnospiraceae NK4A136* and decreased the abundance of *HT002* compared to the other two groups. Linear discriminant analysis (LDA) effect size (LEfSe) and LDA were used to screen for significantly different bacteria in ApoE^−/−^ mice. The Model group exhibited a significant enrichment of *Akkermansia* and *Lactobacillus*, whereas the Capsiate group showed an increase in the abundance of *Desulfovibrio*, *Lachnospiraceae NK4A136*, and *Alloprevotella* (*P* ˂ 0.05, Fig. 9D and E). These results point to the fact that capsiate treatment can reconstruct the intestinal flora, particularly by enhancing the abundance of *Lachnospiraceae NK4A136*.

## DISCUSSION

Atherosclerotic CVD represents a significant contributor to global morbidity and mortality (32). The primary risk factors associated with CVDs have been well established and include hypertension, diabetes mellitus, fluctuations in body weight, and dyslipidemia (36). AS, as a chronic condition, is characterized by endothelial dysfunction, the deposition of lipids and fibrous components within the arterial intima, as well as inflammation, autophagy, and cellular proliferation and migration (2, 32). The predominant pharmacological interventions for atherosclerosis in clinical settings are largely chemical agents, with statins being the most widely utilized due to their effective cholesterol-lowering properties and well-defined mechanisms of action (4, 5). Nonetheless, the administration of statins has been linked to severe adverse effects, including cerebral hemorrhage, renal failure, and rhabdomyolysis (5). The consumption of red pepper and its active component, capsaicin, has been shown to enhance energy and lipid metabolism, potentially through the stimulation of catecholamine release from the adrenal medulla via sympathetic nervous system activation (23). Capsaicin possesses antioxidant, anticancer, and antiangiogenic properties; however, its clinical application has been limited due to its intense pungency, which can cause irritation. In contrast, capsiate, a non-pungent analog of capsaicin, exhibits similar biological effects without the associated irritation and has demonstrated anti-inflammatory and antiangiogenic properties (24, 28, 31). Consequently, it is crucial to investigate the effects of capsiate on AS and the underlying mechanisms involved. In this study, we provide evidence that capsiate treatment significantly reduces atherosclerotic plaque area and exerts a robust anti-atherosclerotic effect in high-fat diet-fed ApoE^−/−^ mice. This effect is mediated through the inhibition of inflammatory responses, regulation of lipid metabolism, and suppression of ferroptosis. Additionally, capsiate appears to positively influence the onset and progression of atherosclerosis by modulating the gut microbiota. The mechanism studies showed that capsiate inhibits the PI3K/AKT/NF-κB pathway while activating the TRPV1 and Nrf2/GPX4/SLC7A11 signaling pathway.

Inflammation is a key feature of AS progression. Numerous studies have indicated that various pathways contribute to the inflammatory response, notably the nuclear translocation of the NF-κB p65 signaling pathway and the PI3K/AKT signaling pathway (7, 9). Upon activation, NF-κB translocates to the nucleus, where the phosphorylated form of NF-κB acts as a transcription factor for inflammatory genes. Furthermore, prior research has established that the activation of the PI3K/AKT pathway plays a role in the activation of NF-κB through the phosphorylation of the IκBα protein. Inhibition of the PI3K/AKT/NF-κB pathway has been proposed as a potential mechanism for anti-AS therapies (8). Here, we found that capsiate could effectively reduce TNF-α, IL-6, and IL-1β levels, while simultaneously increasing the level of IL-10. This effect was achieved through the inhibition of the PI3K/AKT/NF-κB signaling pathway in ApoE^−/−^ mice subjected to a high-fat diet. TRPV1 was initially identified as being expressed in primary nociceptive sensory neurons. However, increasing evidence indicates that TRPV1 activation can improve AS through a variety of mechanisms (25, 33). Consistent with prior studies, capsaicin-induced TRPV1 activation reduces vascular lipid deposition and attenuates AS. Our study further revealed that capsiate can also elevate TRPV1 protein expression in ApoE^−/−^ mice.

Elevated plasma cholesterol levels, particularly high concentrations of LDL-C and non-high-density lipoprotein cholesterol, are significant risk factors for the development of atherosclerosis. The clearance of plasma LDL is facilitated by its binding to LDLR, a process that is regulated by the expression of PCSK9. PCSK9 interacts with LDLR, leading to the internalization and subsequent degradation of LDLR. Additionally, HMGCR serves as the rate-limiting enzyme of hepatic cholesterol synthesis (37). CYP7A1 is the initial rate-limiting enzyme in the conversion of TC into bile acid, and its transcription is positively regulated by liver X receptor α, a member of the nuclear receptor superfamily. A reduction in CYP7A1 activity impedes the conversion of cholesterol to bile acids, resulting in elevated cholesterol levels (38). As presented in this study, the levels of TC, TG, and LDL-C in the ApoE^−/−^ mice fed a high-fat diet were obviously increased. However, capsiate treatment reversed the TC, TG, and LDL-C levels. Fortunately, we also observed that capsiate increased the expression of CYP7A1 and decreased the expression of HMGCR and LDLR in ApoE^−/−^ mice. Collectively, these findings indicated that capsiate may alleviate the progression of AS by modulating lipid metabolism.

Ferroptosis, a novel form of programmed cell death characterized by iron-dependent lipid peroxidation, was first proposed by Dixon in 2012 (17). This process is initiated by an imbalance in cellular metabolism and redox homeostasis, and it can be inhibited through direct intervention in lipid peroxidation or via pharmacological or genetic strategies aimed at iron depletion. AS is associated with iron-dependent cell death resulting from oxidative stress (16). Prior research has established that lipid peroxidation significantly contributes to the pathogenesis of atherosclerosis by inducing inflammation and endothelial dysfunction, while lipid peroxidation is the core feature of ferroptosis, which plays an important role in atherosclerosis (19, 34). Moreover, ferroptosis has been recognized as a critical molecular mechanism underlying the pharmacological actions of cardiovascular protective agents and their active components. GPX4 serves as a vital protector against ferroptosis by converting harmful lipid hydroperoxides into non-toxic lipid alcohols, thereby mitigating lipid peroxidation (18). SLC7A11 is an essential subunit of system Xc- that regulates the reverse transport of glutamate and cystine (39). Nrf2 is a key antioxidant transcription factor that plays a significant role in sustaining redox and metabolic homeostasis by regulating cellular antioxidants, including the expression of SLC7A11 and GPX4 (34, 40). In the context of AS, the Nrf2/GPX4/SLC7A11 signaling pathway has been implicated in the regulation of ferroptosis in endothelial cells (41). Previous studies have identified that capsiate is a potent inhibitor of ferroptosis (31). Consequently, we investigated whether the protective effects of capsiate against AS are linked to its anti-ferroptotic mechanisms. Our findings indicate a significant increase in ferroptosis-related markers within the aortas of ApoE^−/−^ mice fed high-fat diet. Moreover, we observed that capsiate effectively reversed the changes in ferroptosis markers induced by the high-fat diet *in vivo*. Mechanistically, our data suggest that capsiate not only enhances Nrf2 expression but also elevates the protein levels of GPX4 and SLC7A11. Furthermore, it was also observed that capsiate inhibited ox-LDL-induced cell injury, inflammatory response, and ferroptosis in HUVECs. The role of Nrf2 in ox-LDL-induced ferroptosis was further validated using ML385, a specific Nrf2 antagonist, which demonstrated that ML385 negated the inhibitory effects of capsiate on ferroptosis in HUVECs. Thus, it can be concluded that capsiate may mitigate atherosclerosis by modulating ferroptosis through the Nrf2/GPX4/SLC7A11 pathway.

Research has demonstrated that a high-fat diet affects the diversity and composition of the gut microbiota in both humans and rodents. Given that a high-fat diet can alter the composition of the gut microbiota (11), this study aimed to examine the impact of capsiate on the composition and diversity of the gut microbiota in ApoE^−/−^ mice fed a high-fat diet. To evaluate the effects of capsiate on the gut microbiota composition and diversity in association with high-fat diet feeding, we performed 16S rRNA sequencing and analyzed the data using various bioinformatics tools. In this study, we observed that the Model group exhibited a reduced diversity of the microbiome, as indicated by the Observed_species, Shannon, Simpson, Chao1, ACE and Pielou_e index. Conversely, capsiate treatment appeared to enhance gut microbiota diversity, as indicated by increased α-diversity. The analysis of β-diversity, utilizing PCA and NMDS, revealed a significant distinction in the gut microbiota structure between the Control and Model groups. In contrast, the Control group and Capsiate group demonstrated a high degree of intergroup similarity and relatively similar community composition. A healthy gut microbiota is characterized by a predominance of anaerobic *Firmicutes* and *Bacteroidetes*, which collectively account for approximately 90% of the microbiome. The ratio of *Firmicutes*/*Bacteroidetes* is considered as one of the biomarkers for measuring the changes in microbial community structure (35). Our findings indicate that capsiate treatment significantly reduced the ratio of *Firmicutes*/*Bacteroidetes*. Additionally, at the genus level, capsiate significantly increased the abundance of the probiotics *Ligilactobacillus*, *Lachnospiraceae NK4A136* group, and *Muribaculum*. Recent research has demonstrated that α-mangostin mitigates ethanol-induced gastric ulceration while concurrently increasing the abundance of *Ligilactobacillus* and *Muribaculum* (42). Studies have demonstrated that an increase in the abundance of the *Lachnospiraceae NK4A136* group in mouse feces has been linked to lower glucose concentrations, improved glucose tolerance, and reduced inflammation (43). Furthermore, the supplementation of *Lactobacillus reuteri CCFM8631* has been shown to mitigate atherosclerotic CVD through the modulation of gut microbiota, as indicated by an increase in the relative abundance of the *Lachnospiraceae NK4A136* group (44). Therefore, it is reasonable to speculate that the reduction of high-fat diet-induced atherosclerosis by capsiate may be partially attributed to the enhancement of gut microbiota diversity and the abundance of probiotics.

All of these findings suggest the capsiate can inhibit AS and hold promise for clinical applications. However, it is essential to investigate its pharmacokinetic properties and identify the patient populations that would benefit from its use. Addressing these inquiries will require substantial research efforts. Furthermore, while some advancements have been achieved, the clinical utilization of TRPV1 in the prevention of atherosclerosis necessitates further investigation.

### Conclusion

**Fig 10:**
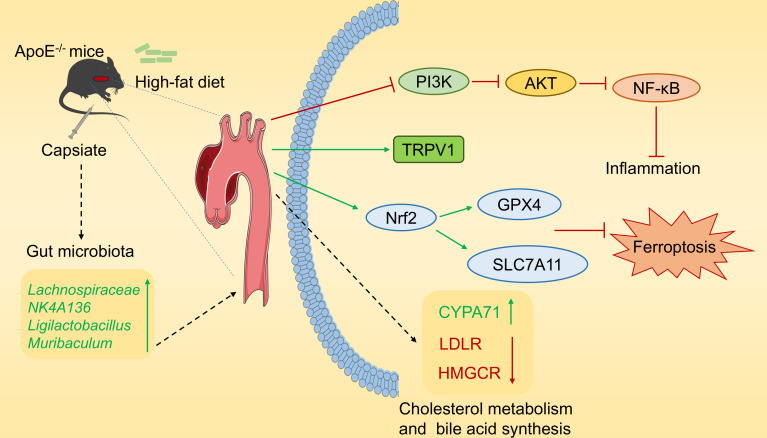
Graphical illustration of the molecular mode of capsiate protecting against atherosclerosis.

In summary, this research investigated the impact of capsiate administration on the development of AS in ApoE^−/−^ mice fed a high-fat diet (Fig. 10). The findings indicate that capsiate administration contributed to ameliorate AS-related phenotypes, including alterations in lipid metabolism, inflammation, and ferroptosis. The protective effects of capsiate against atherosclerosis may be mediated through the PI3K/AKT/NF-κB and Nrf2/GPX4/SLC7A11 signaling pathways. In addition, capsiate can restructure the gut microbiota in AS mice. These findings demonstrate the potential application of capsiate as a novel therapeutic agent for the prevention of AS and provide a novel approach for addressing this CVD.

## SUPPLEMENTAL MATERIAL

Table S1 spectrum.03155-24-s0001.docx Primer sequences for quantitative real-time PCR.

ASM does not own the copyrights to Supplemental Material that may be linked to, or accessed through, an article. The authors have granted ASM a non-exclusive, world-wide license to publish the Supplemental Material files. Please contact the corresponding author directly for reuse.
