# Supplementary material for: Capsiate attenuates atherosclerosis by activating Nrf2/GPX4 pathway and reshaping the intestinal microbiota in ApoE−/− mice
Source: Microbiol Spectr. 2025 Mar 3;13(4):e03155-24. doi: 10.1128/spectrum.03155-24 (PMC11960139; doi:10.1128/spectrum.03155-24)
Supplement: Table S1 — Primer sequences for quantitative real-time PCR. [file spectrum.03155-24-s0001.docx]

**Supplementary material**

**Table S1** Primer sequences for quantitative real-time PCR.

| Species | Primers | Forward Sequences (5′→3′) | Reverse Sequences (5′→3′) |
| --- | --- | --- | --- |
| Human | IL-6 | ACAGCCACTCACCTCTTCAG | CCATCTTTTTCAGCCATCTTT |
|  | IL-1β | TGCCACCTTTTGACAGTGATG | AAGGTCCACGGGAAAGACAC |
|  | TNF-α | GCTGCACTTTGGAGTGATCG | ATGAGGTACAGGCCCTCTGA |
|  | Nrf2 | TCTGGAAAGGACCGTTGTCG | GCCAAGTAGTGTGTCTCCATAG |
|  | GPX4 | AGAGATCAAAGAGTTCGCCG | TTGTCGATGAGGAACTGTGG |
|  | SLC7A11 | GCGTGGGCATGTCTCTGAC | GCTGGTAATGGACCAAAGACTTC |
|  | GAPDH | GGTCGGAGTCAACGGATTTGGTCG | CCTCCGACGCCTGCTTCACCAC |
